# Dimethyl­ammonium diaqua­(pyridine-2,4-dicarboxyl­ato-κ^2^ *N*,*O* ^2^)cuprate(II)

**DOI:** 10.1107/S1600536810002497

**Published:** 2010-01-27

**Authors:** Ji-Dong Wang, Shu-Min Han

**Affiliations:** a College of Environmental and Chemical Engineering, Yanshan University, Qinhuangdao 066004, People’s Republic of China; b College of Information Technology and Engineering, Yanshan University, Qinhuangdao 066004, People’s Republic of China; c State Key Laboratory of Metastable Materials Science and Technology, Yanshan University, Qinhuangdao 066004, People’s Republic of China

## Abstract

The asymmetric unit of the title compound, (C_2_H_8_N)_2_[Cu(C_7_H_3_NO_4_)_2_(H_2_O)_2_], contains one-half of a mononuclear [Cu(C_7_H_3_NO_4_)_2_(H_2_O)_2_]^2−^ anion, one dimethyl­ammonium cation and one aqua ligand. The Cu^II^ atom, lying on an inversion center, is coordinated by two symmetry-related N atoms and two O atoms from one pyridine-2,4-dicarboxyl­ate ligand and two symmetry-related aqua ligands and exhibits a distorted octa­hedral *trans*-[CuN_2_O_4_] coordination geometry. Multiple crystallographically independent O—H⋯O and N—H⋯O hydrogen bonds form a three-dimensional network in the crystal structure.

## Related literature

For the structural diversity and potential applications of coordination polymers constructed from metal ions and bridging ligands, see: Eddaoudi *et al.* (2001 ▶); Kitagawa *et al.* (2004 ▶). For general background to metal complexes of pyridine-2,4-dicarboxyl­ates, see: Mahata & Natarajan (2005 ▶); Bai *et al.* (2008 ▶); Chen & Beatty (2008 ▶). For similar structures, see: Zou *et al.* (2008 ▶); Noro *et al.* (2005 ▶). For comparative bond lengths and angles, see: Chutia *et al.* (2009 ▶); Klein *et al.* (1982 ▶).
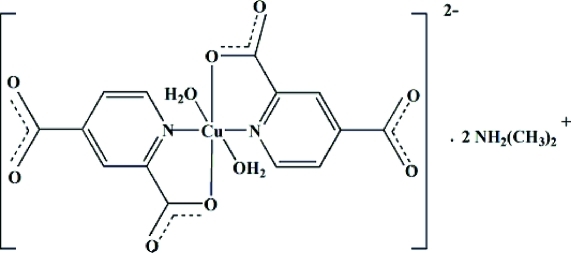


## Experimental

### 

#### Crystal data

(C_2_H_8_N)_2_[Cu(C_7_H_3_NO_4_)_2_(H_2_O)_2_]
*M* *_r_* = 521.98
Monoclinic, 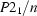

*a* = 7.9854 (7) Å
*b* = 9.4648 (8) Å
*c* = 14.9380 (12) Å
β = 103.540 (1)°
*V* = 1097.64 (16) Å^3^
*Z* = 2
Mo *K*α radiation
μ = 1.06 mm^−1^
*T* = 293 K
0.31 × 0.16 × 0.16 mm

#### Data collection

Bruker SMART APEX CCD diffractometer
Absorption correction: multi-scan (*SADABS*; Sheldrick, 1996 ▶) *T*_min_ = 0.732, *T*_max_ = 0.849
5508 measured reflections
2160 independent reflections
1992 reflections with *I* > 2σ(*I*)
*R*_int_ = 0.016

#### Refinement

*R*[*F* ^2^ > 2σ(*F* ^2^)] = 0.029
*wR*(*F* ^2^) = 0.079
*S* = 1.06
2160 reflections
159 parameters
2 restraints
H atoms treated by a mixture of independent and constrained refinement
Δρ_max_ = 0.35 e Å^−3^
Δρ_min_ = −0.26 e Å^−3^

### 

Data collection: *SMART* (Bruker, 2007 ▶); cell refinement: *SAINT* (Bruker, 2007 ▶); data reduction: *SAINT*; program(s) used to solve structure: *SHELXS97* (Sheldrick, 2008 ▶); program(s) used to refine structure: *SHELXL97* (Sheldrick, 2008 ▶); molecular graphics: *SHELXTL* (Sheldrick, 2008 ▶); software used to prepare material for publication: *SHELXTL*.

## Supplementary Material

Crystal structure: contains datablocks global, I. DOI: 10.1107/S1600536810002497/bx2257sup1.cif

Structure factors: contains datablocks I. DOI: 10.1107/S1600536810002497/bx2257Isup2.hkl

Additional supplementary materials:  crystallographic information; 3D view; checkCIF report

## Figures and Tables

**Table 1:** Selected bond lengths (Å)

Cu1—O1	1.9733 (11)
Cu1—N1	1.9810 (14)
Cu1—O1*W*	2.4162 (15)

**Table 2:** Hydrogen-bond geometry (Å, °)

*D*—H⋯*A*	*D*—H	H⋯*A*	*D*⋯*A*	*D*—H⋯*A*
O1*W*—H1*WA*⋯O4^i^	0.83 (2)	1.85 (2)	2.680 (2)	174
O1*W*—H1Wb⋯O3^ii^	0.81 (2)	2.00 (2)	2.809 (2)	172
N2—H2*A*⋯O3	0.90	1.92	2.783 (2)	161
N2—H2*B*⋯O2^iii^	0.90	1.94	2.778 (2)	154

Symmetry codes: (i) 
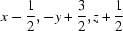
; (ii) 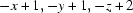; (iii) 
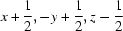
.

**Fig. 1.**
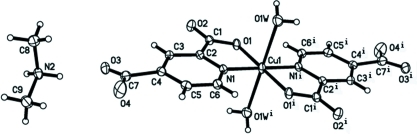
Molecular structure of the title compound. Displacement ellipsoids are drawn at the 30% probability level. [Symmetry code: (i) -x, 1 - y, 2 - z.]

## supplementary crystallographic information

### Comment

Coordination polymers constructed from metal ions and bridging ligands have been of great interest due to their structural diversity and many potential applications (Eddaoudi *et al.*, 2001; Kitagawa *et al.*, 2004). Pyridinedicarboxylates(pydc) have been extensively studied as excellent bridging ligands in the area of metal-organic frameworks (Mahata *et al.*, 2005; Bai *et al.* 2008; Chen *et al.* 2008). Herein we report the crystal structure of the title compound [Cu(2,4-pydc)_2_(H_2_O)_2_][NH_2_(CH_3_)_2_]_2_, (2,4-pydc= pyridine-2,4-dicarboxylate) .The Cu^II^ atom, lying on an inversion center, is coordinated by two symmetry-related N atoms and two O atoms from one pyridine-2,4-dicarboxylate ligand and two symmetry-related aqua ligands and exhibits a distorted octahedral trans-[ CuN_2_O_4_] coordination geometry (Table 1 and Fig. 1). The bond lengths and angles are all in normal ranges (Chutia *et al.*, 2009; Klein *et al.*, 1982). Multiple crystallographically independent hydrogen bonds form a three-dimensional network in the crystal structure, Table 2.

### Experimental

A solution of Cu(NO_3_)_2_.3H_2_O (0.024 g, 0.1 mmol) in H_2_O (3 ml) was added to a suspending solution of 2,4-pydc (0.017 g, 0.1 mmol) in H_2_O and DMF(1:1, 7 ml). The mixture was stirred for 30 minutes and sealed in a 15 ml Teflon-lined stainless steel autoclave and heated at 423 K for 3 d under autogenous pressure. When cooled to room temperature, green block crystals of the title compound were obtained (yield 0.045 g, 86% based on Cu).

### Refinement

H atoms of the pyridine ring were positioned geometrically and refined as riding atoms, with C—H = 0.93-0.96 Å and with *U*_iso_(H) = 1.2*U*_eq_(C) or 1.5 U_eq_(C) for CH_3 _group. H atoms of water molecule were located in a difference Fourier map and refined as riding, with *U*_iso_(H) = 1.2*U*_eq_(O).

### Crystal data

**Table:**

(C_2_H_8_N)_2_[Cu(C_7_H_3_NO_4_)_2_(H_2_O)_2_]	*F*(000) = 542
*M**_r_* = 521.98	*D*_x_ = 1.579 Mg m^−^^3^
Monoclinic, *P*2_1_/*n*	Mo *K*α radiation, λ = 0.71073 Å
Hall symbol: -P 2yn	Cell parameters from 3343 reflections
*a* = 7.9854 (7) Å	θ = 2.6–26.0°
*b* = 9.4648 (8) Å	µ = 1.06 mm^−^^1^
*c* = 14.9380 (12) Å	*T* = 293 K
β = 103.540 (1)°	Block, green
*V* = 1097.64 (16) Å^3^	0.31 × 0.16 × 0.16 mm
*Z* = 2	

### Data collection

**Table:**

Bruker SMART APEX CCD diffractometer	2160 independent reflections
Radiation source: sealed tube	1992 reflections with *I* > 2σ(*I*)
graphite	*R*_int_ = 0.016
φ and ω scans	θ_max_ = 26.0°, θ_min_ = 2.6°
Absorption correction: multi-scan (*SADABS*; Sheldrick, 1996)	*h* = −9→9
*T*_min_ = 0.732, *T*_max_ = 0.849	*k* = −7→11
5508 measured reflections	*l* = −12→18

### Refinement

**Table:**

Refinement on *F*^2^	Primary atom site location: structure-invariant direct methods
Least-squares matrix: full	Secondary atom site location: difference Fourier map
*R*[*F*^2^ > 2σ(*F*^2^)] = 0.029	Hydrogen site location: inferred from neighbouring sites
*wR*(*F*^2^) = 0.079	H atoms treated by a mixture of independent and constrained refinement
*S* = 1.06	*w* = 1/[σ^2^(*F*_o_^2^) + (0.0456*P*)^2^ + 0.427*P*] where *P* = (*F*_o_^2^ + 2*F*_c_^2^)/3
2160 reflections	(Δ/σ)_max_ = 0.001
159 parameters	Δρ_max_ = 0.35 e Å^−^^3^
2 restraints	Δρ_min_ = −0.26 e Å^−^^3^

### Fractional atomic coordinates and isotropic or equivalent isotropic displacement parameters (Å^2^)

**Table:**

	*x*	*y*	*z*	*U*_iso_*/*U*_eq_	
Cu1	0.0000	0.5000	1.0000	0.02668 (12)	
N1	0.15726 (18)	0.55054 (15)	0.92027 (9)	0.0235 (3)	
N2	0.7815 (2)	0.47684 (16)	0.59707 (11)	0.0302 (3)	
H2A	0.6957	0.4670	0.6264	0.036*	
H2B	0.8078	0.3904	0.5793	0.036*	
O1	0.15272 (15)	0.33417 (12)	1.02961 (8)	0.0291 (3)	
O2	0.38987 (16)	0.24074 (13)	0.99920 (9)	0.0337 (3)	
O3	0.57116 (18)	0.46822 (17)	0.72146 (10)	0.0393 (3)	
O4	0.4794 (2)	0.68865 (19)	0.68723 (12)	0.0618 (5)	
O1W	0.18306 (19)	0.62993 (16)	1.12457 (10)	0.0400 (3)	
H1WA	0.126 (3)	0.687 (2)	1.1473 (16)	0.048*	
H1WB	0.250 (2)	0.594 (2)	1.1680 (12)	0.048*	
C1	0.2761 (2)	0.33162 (18)	0.98819 (11)	0.0253 (3)	
C2	0.2792 (2)	0.45160 (18)	0.92182 (11)	0.0223 (3)	
C3	0.3928 (2)	0.45788 (18)	0.86496 (11)	0.0234 (3)	
H3	0.4755	0.3880	0.8674	0.028*	
C4	0.3812 (2)	0.57093 (18)	0.80377 (11)	0.0249 (3)	
C5	0.2608 (2)	0.67552 (18)	0.80637 (11)	0.0277 (4)	
H5	0.2545	0.7549	0.7690	0.033*	
C6	0.1501 (2)	0.66179 (18)	0.86455 (11)	0.0269 (4)	
H6	0.0686	0.7319	0.8649	0.032*	
C7	0.4887 (2)	0.5772 (2)	0.73205 (12)	0.0337 (4)	
C8	0.9339 (3)	0.5361 (3)	0.66186 (15)	0.0414 (5)	
H8A	1.0242	0.5513	0.6303	0.062*	
H8B	0.9728	0.4712	0.7118	0.062*	
H8C	0.9037	0.6244	0.6856	0.062*	
C9	0.7204 (3)	0.5659 (2)	0.51421 (13)	0.0409 (5)	
H9A	0.6653	0.6490	0.5306	0.061*	
H9B	0.6396	0.5134	0.4686	0.061*	
H9C	0.8166	0.5931	0.4898	0.061*	

### Atomic displacement parameters (Å^2^)

**Table:**

	*U*^11^	*U*^22^	*U*^33^	*U*^12^	*U*^13^	*U*^23^
Cu1	0.03408 (19)	0.02407 (18)	0.02744 (19)	0.00826 (11)	0.01843 (13)	0.00608 (11)
N1	0.0288 (7)	0.0230 (7)	0.0207 (6)	0.0025 (6)	0.0095 (5)	0.0003 (6)
N2	0.0352 (8)	0.0289 (8)	0.0295 (8)	−0.0024 (6)	0.0136 (7)	−0.0061 (6)
O1	0.0363 (7)	0.0269 (6)	0.0297 (6)	0.0071 (5)	0.0190 (5)	0.0078 (5)
O2	0.0357 (7)	0.0289 (7)	0.0411 (7)	0.0097 (5)	0.0183 (6)	0.0119 (6)
O3	0.0368 (7)	0.0531 (8)	0.0329 (7)	0.0035 (6)	0.0183 (6)	0.0039 (6)
O4	0.0617 (10)	0.0642 (11)	0.0720 (11)	0.0089 (8)	0.0411 (9)	0.0378 (9)
O1W	0.0444 (8)	0.0413 (8)	0.0345 (7)	0.0109 (6)	0.0095 (6)	−0.0020 (6)
C1	0.0304 (8)	0.0230 (8)	0.0242 (8)	0.0016 (7)	0.0097 (7)	0.0016 (6)
C2	0.0258 (8)	0.0208 (8)	0.0209 (7)	−0.0009 (6)	0.0070 (6)	−0.0010 (6)
C3	0.0236 (8)	0.0235 (8)	0.0240 (8)	0.0001 (6)	0.0074 (6)	−0.0001 (7)
C4	0.0245 (8)	0.0276 (9)	0.0228 (8)	−0.0058 (6)	0.0062 (6)	0.0009 (7)
C5	0.0333 (9)	0.0245 (9)	0.0250 (8)	−0.0027 (7)	0.0063 (7)	0.0059 (7)
C6	0.0320 (8)	0.0231 (8)	0.0260 (8)	0.0043 (7)	0.0079 (7)	0.0026 (7)
C7	0.0277 (9)	0.0459 (12)	0.0287 (9)	−0.0047 (8)	0.0090 (7)	0.0085 (8)
C8	0.0349 (10)	0.0507 (12)	0.0376 (11)	−0.0059 (9)	0.0066 (8)	−0.0036 (10)
C9	0.0472 (11)	0.0445 (12)	0.0319 (10)	0.0041 (9)	0.0110 (8)	0.0005 (9)

### Geometric parameters (Å, °)

**Table:**

Cu1—O1	1.9733 (11)	O1W—H1WB	0.815 (10)
Cu1—O1^i^	1.9733 (11)	C1—C2	1.512 (2)
Cu1—N1^i^	1.9810 (14)	C2—C3	1.381 (2)
Cu1—N1	1.9810 (14)	C3—C4	1.396 (2)
Cu1—O1W^i^	2.4162 (15)	C3—H3	0.9300
Cu1—O1W	2.4162 (15)	C4—C5	1.387 (2)
N1—C6	1.335 (2)	C4—C7	1.523 (2)
N1—C2	1.347 (2)	C5—C6	1.383 (2)
N2—C8	1.477 (3)	C5—H5	0.9300
N2—C9	1.483 (3)	C6—H6	0.9300
N2—H2A	0.9000	C8—H8A	0.9600
N2—H2B	0.9000	C8—H8B	0.9600
O1—C1	1.281 (2)	C8—H8C	0.9600
O2—C1	1.234 (2)	C9—H9A	0.9600
O3—C7	1.253 (2)	C9—H9B	0.9600
O4—C7	1.242 (2)	C9—H9C	0.9600
O1W—H1WA	0.832 (10)		
			
O1—Cu1—O1^i^	179.998 (1)	N1—C2—C3	122.38 (15)
O1—Cu1—N1^i^	96.81 (5)	N1—C2—C1	114.25 (14)
O1^i^—Cu1—N1^i^	83.18 (5)	C3—C2—C1	123.33 (15)
O1—Cu1—N1	83.19 (5)	C2—C3—C4	118.89 (16)
O1^i^—Cu1—N1	96.81 (5)	C2—C3—H3	120.6
N1^i^—Cu1—N1	180.00 (5)	C4—C3—H3	120.6
O1—Cu1—O1W^i^	89.92 (5)	C5—C4—C3	117.97 (15)
O1^i^—Cu1—O1W^i^	90.08 (5)	C5—C4—C7	120.04 (15)
N1^i^—Cu1—O1W^i^	89.18 (5)	C3—C4—C7	121.90 (16)
N1—Cu1—O1W^i^	90.82 (5)	C6—C5—C4	119.98 (15)
O1—Cu1—O1W	90.08 (5)	C6—C5—H5	120.0
O1^i^—Cu1—O1W	89.91 (5)	C4—C5—H5	120.0
N1^i^—Cu1—O1W	90.82 (5)	N1—C6—C5	121.69 (15)
N1—Cu1—O1W	89.18 (5)	N1—C6—H6	119.2
O1W^i^—Cu1—O1W	180.00 (5)	C5—C6—H6	119.2
C6—N1—C2	118.96 (14)	O4—C7—O3	126.71 (18)
C6—N1—Cu1	128.65 (12)	O4—C7—C4	116.09 (18)
C2—N1—Cu1	112.29 (11)	O3—C7—C4	117.13 (16)
C8—N2—C9	113.01 (16)	N2—C8—H8A	109.5
C8—N2—H2A	109.0	N2—C8—H8B	109.5
C9—N2—H2A	109.0	H8A—C8—H8B	109.5
C8—N2—H2B	109.0	N2—C8—H8C	109.5
C9—N2—H2B	109.0	H8A—C8—H8C	109.5
H2A—N2—H2B	107.8	H8B—C8—H8C	109.5
C1—O1—Cu1	114.32 (10)	N2—C9—H9A	109.5
Cu1—O1W—H1WA	110.7 (17)	N2—C9—H9B	109.5
Cu1—O1W—H1WB	124.4 (18)	H9A—C9—H9B	109.5
H1WA—O1W—H1WB	106 (2)	N2—C9—H9C	109.5
O2—C1—O1	125.07 (15)	H9A—C9—H9C	109.5
O2—C1—C2	119.13 (14)	H9B—C9—H9C	109.5
O1—C1—C2	115.80 (14)		

Symmetry codes: (i) −*x*, −*y*+1, −*z*+2.

### Hydrogen-bond geometry (Å, °)

**Table:**

*D*—H···*A*	*D*—H	H···*A*	*D*···*A*	*D*—H···*A*
O1W—H1WA···O4^ii^	0.83 (2)	1.85 (2)	2.680 (2)	174
O1W—H1Wb···O3^iii^	0.812 (18)	2.002 (17)	2.809 (2)	172
N2—H2A···O3	0.90	1.92	2.783 (2)	161
N2—H2B···O2^iv^	0.90	1.94	2.778 (2)	154

Symmetry codes: (ii) *x*−1/2, −*y*+3/2, *z*+1/2; (iii) −*x*+1, −*y*+1, −*z*+2;  (iv) *x*+1/2, −*y*+1/2, *z*−1/2.
